# Right Ventricular Lipoma With an Aneurysm‐Like Appearance: A Case Report of Successful Surgical Resection

**DOI:** 10.1155/cris/4331944

**Published:** 2026-08-30

**Authors:** Yuta Inoue, Takayoshi Kato, Ryo Fujii, Mizuki Kadota, Natsuko Suzui, Masayuki Sato, Hiroki Ogura, Etsuji Umeda, Osamu Sakai, Kiyoshi Doi

**Affiliations:** ^1^ Department of Cardiovascular Surgery, Gifu University Hospital, Gifu, Japan, gifu-u.ac.jp; ^2^ Department of Pathology, Gifu University Hospital, Gifu, Japan, gifu-u.ac.jp

**Keywords:** cardiac lipoma, case reports, elevated right-sided pressures, right ventricle

## Abstract

Cardiac lipomas are rare benign tumors, comprising ~8.4% of all benign primary cardiac tumors. They can arise in various cardiac chambers, though right ventricular (RV) involvement is uncommon. While most cardiac lipomas are asymptomatic and detected incidentally, those originating in the RV may present with unusual features, including pseudoaneurysmal appearances. Such anomalous structural changes can increase right atrial and ventricular pressures, placing the patient at risk of progressive hemodynamic compromise and rupture. We report a case of an RV lipoma initially diagnosed as a pseudoaneurysm that was successfully resected. An 81‐year‐old man with an incidentally detected RV mass was admitted to our hospital. Contrast‐enhanced computed tomography (CT) showed that the mass contained a large amount of fatty tissue with a pseudoaneurysmal appearance, suggesting it to be an RV lipoma. Transthoracic echocardiography revealed to‐and‐fro blood flow between the RV and the aneurysm through the slits. Although the patient was asymptomatic, considering that the right atrial pressure and RV end‐diastolic pressure (RVEDP) were 12 and 25 mmHg, respectively, based on right heart catheterization, we decided to perform surgical resection to avoid the risk of future enlargement or rupture. A fat‐covered mass was found in the anterior aspect of the RV and resected. The residual RV and slits were closed using a patch. The patient had a good postoperative course with normalized right heart pressure and was discharged on the postoperative day 14. While the indications for surgery in asymptomatic patients remain unclear due to the rarity of this condition, these findings suggest that early surgical intervention may be considered in cases presenting with structural compromise and elevated right‐sided pressures.

## 1. Introduction

Cardiac lipoma is a relatively rare tumor, accounting for 8.4% of benign primary cardiac tumors, exhibiting a broad range of appearances and sizes [1]. According to a review of 208 cases of cardiac lipomas, only 10 were found in the right ventricle (RV) [2].

Although most cardiac lipomas are asymptomatic and discovered incidentally, RV lipomas may show atypical morphological features, including a pseudoaneurysm‐like appearance [3]. These abnormal structural changes can be associated with elevated right atrial and ventricular pressures and may raise concern for progressive hemodynamic deterioration [3]. Although observation may be appropriate for small, asymptomatic cardiac lipomas without hemodynamic effects, previous reports have suggested that surgical resection can be a reasonable and safe option in selected asymptomatic patients, particularly when the lesion is large, atypical, or diagnostically uncertain [4]. Here, we report a successfully resected case of an RV lipoma with an aneurysm‐like appearance and elevated right‐sided pressures.

## 2. Case Presentation

**Figure 1:**
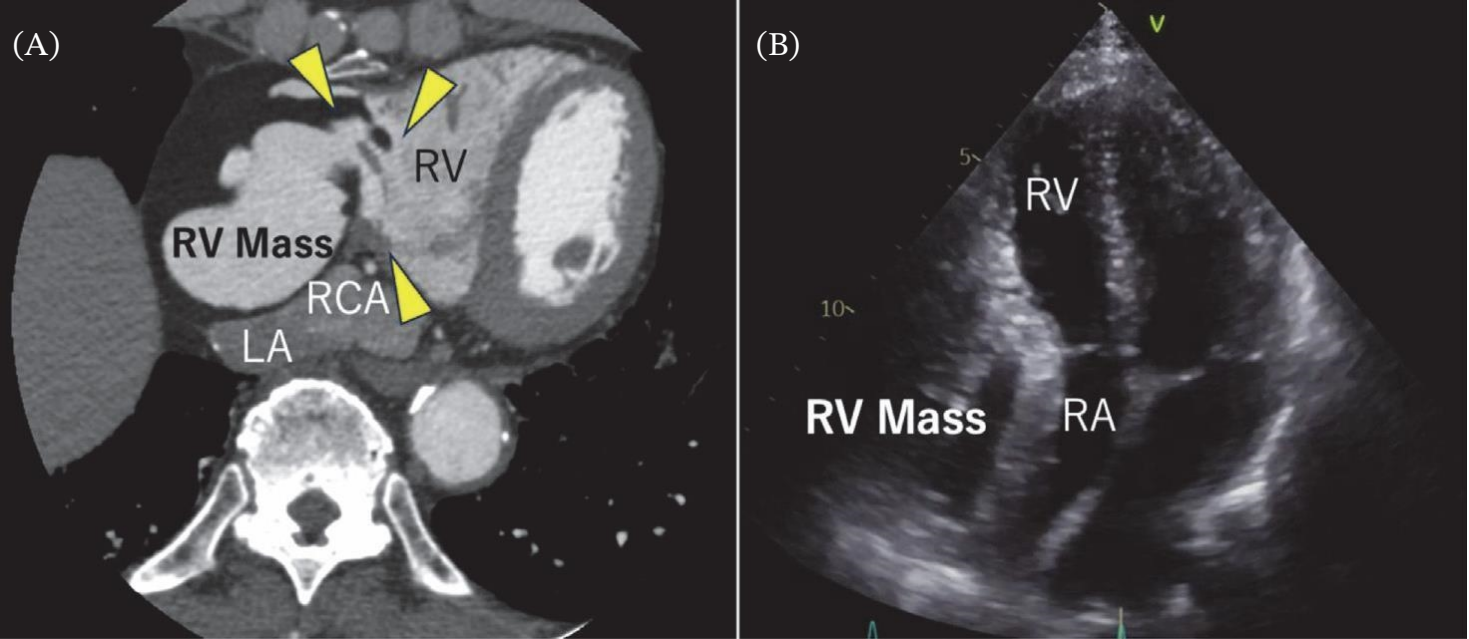
Preoperative imaging. CT revealing a 47 mm × 76 mm mass with multiple slit‐like communications connecting the RV and mass (arrowhead). The predominantly adipose composition of the mass wall suggests a lipoma (A). Transthoracic echocardiography demonstrating that the mass in the RV compressed and deformed the RA (B), with evidence of to‐and‐fro blood flow between the RV and mass. CT, computed tomography; RA, right atrium; RV, right ventricle.

An 81‐year‐old man receiving medications for hypertension and diabetes was referred by a primary care physician for pneumonia. Computed tomography (CT) incidentally detected an abnormal mediastinal mass with a diameter of 47 mm × 76 mm. Contrast‐enhanced CT revealed that the mass had protruded from the RV with a blood‐filled cavity, presenting as an RV aneurysm (Figure 1A). Multiple slit‐like communications were observed between the RV and the mass. We suspected that the mass was a cardiac lipoma because the Hounsfield unit value on the CT image indicated that it was predominantly composed of adipose tissue.

Transthoracic echocardiography showed that the RV mass compressed the right atrium (RA), resulting in RA deformation (Figure 1B) and a to‐and‐fro blood flow between the RV and the mass (Video S1). The mass contracted during the RV diastolic phase and expanded during the systolic phase. Coronary angiography revealed a normal coronary artery. Right heart catheterization revealed that the RA pressure (RAP) and RV end‐diastolic pressure (RVEDP) had increased to 12 and 25 mmHg, respectively.

As the patient had no history of trauma or myocardial infarction, the mechanism by which the mass acquired its aneurysmal appearance in the RV was unknown. Furthermore, the aneurysm had a wall thickness of only 3 mm at the thinnest point and was at risk of rupture. Although the patient was asymptomatic, we decided to perform surgical resection of the mass and reconstruction of the RV. Because of the possibility of tight adhesion between the mass and sternum, a median sternotomy could result in fatal hemorrhage. Therefore, a vascular graft was anastomosed to the right axillary artery for arterial perfusion, a long venous cannula was inserted into the RA via the right femoral vein, and cardiopulmonary bypass was established before sternotomy.

**Figure 2:**
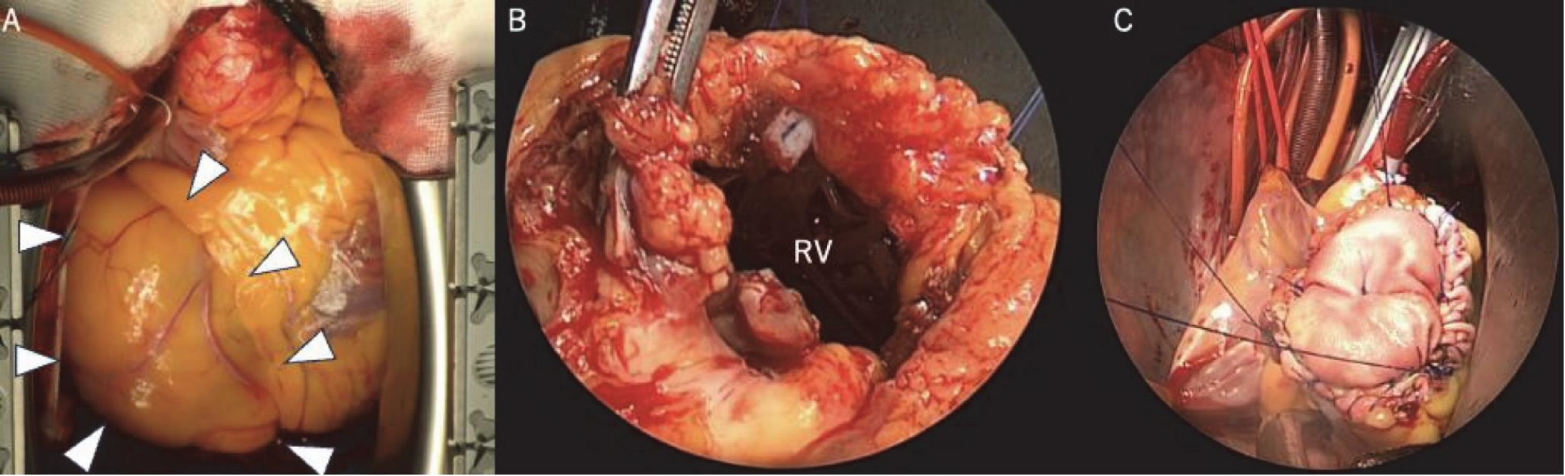
Operative findings. Intraoperative inspection reveals a soft, yellowish, elastic mass composed predominantly of fat, bulging from the anterior free wall of the RV and exhibiting blood flow (A). The fatty mass was completely excised, exposing the normal myocardium of the RV wall, resulting in an ~3‐cm hole in the RV (B). The RV wall was repaired using Sauvage Filamentous Fabric patches (C). RV, right ventricle.

Fortunately, no adhesions were observed between the mass and the pericardium and sternum. A soft, yellowish, elastic, fat‐covered mass bulging around the RV with blood flow was observed in its anterior free wall (Figure 2A). The fatty mass was thoroughly resected until the normal RV wall myocardium was exposed, resulting in an ~3 cm defect in size in the RV (Figure 2B). The RV wall was repaired using Sauvage Filamentous Fabric patches (Bard Peripheral Vascular, Inc., Tempe, AZ, USA) (Figure 2C).

**Figure 3:**
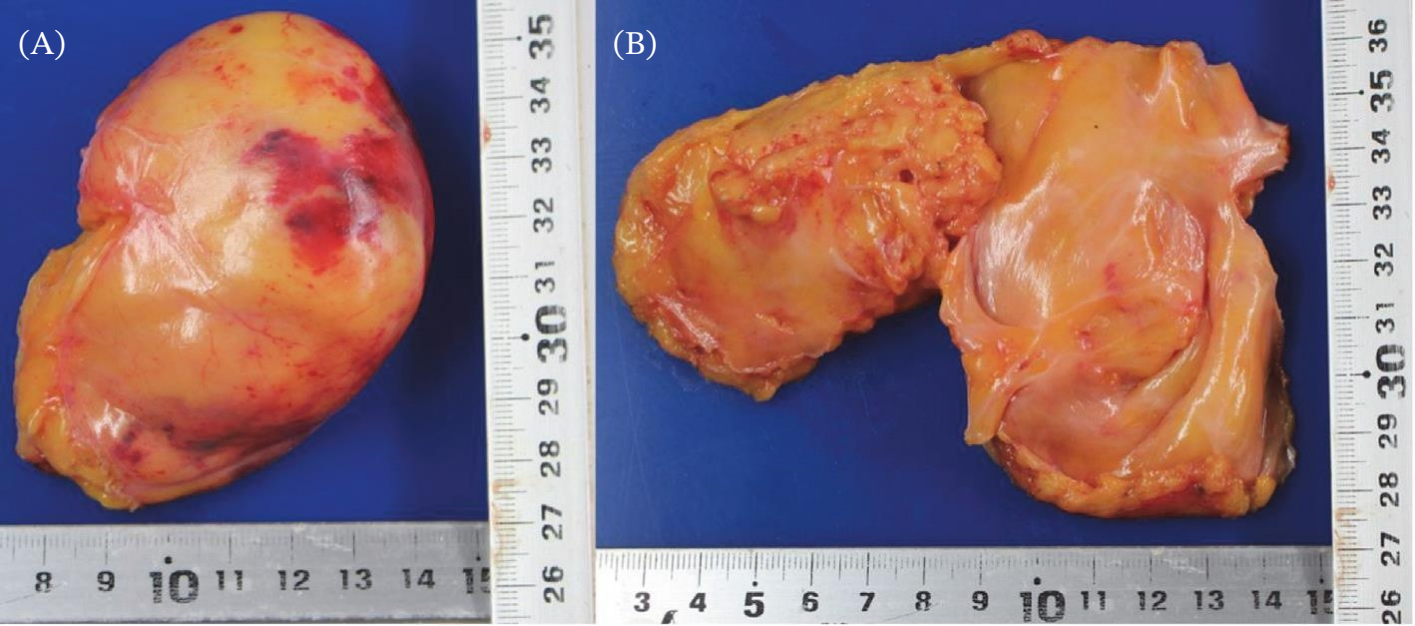
Macroscopic findings of the RV lipoma. The mass is measured 85 mm × 60 mm × 50 mm (A). The inner surface is yellowish and smooth. Some slit‐like structures are observed between the RV and intracavity lumen (B).

The mass was measured to be 85 mm × 60 mm × 50 mm (Figure 3A). The inner surface was yellowish and smooth. Some slit‐like structures were observed between the RV and the intracavity lumen (Figure 3B).

**Figure 4:**
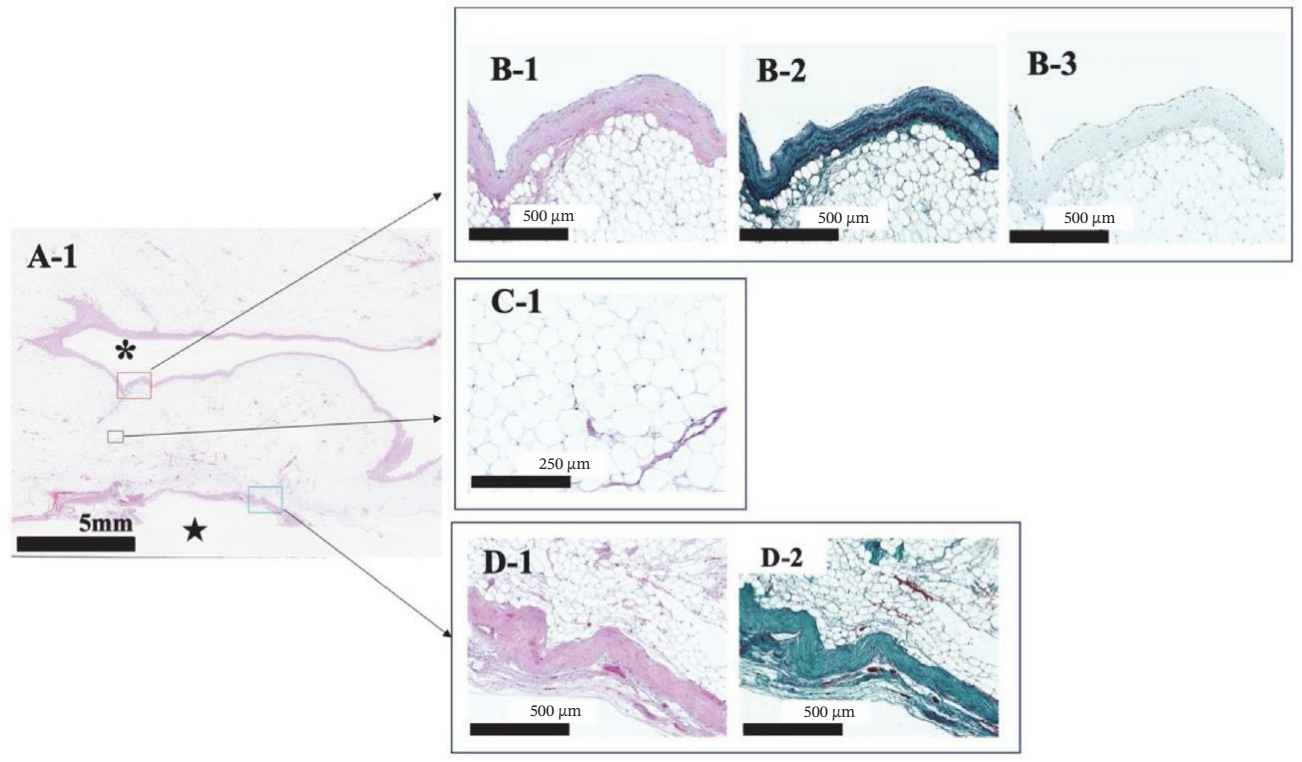
Histological findings. Histopathological examination of the mass performed using hematoxylin and eosin (H&E) and Elastica–Masson (E&M) staining. (A‐1) The wall of the aneurysmal mass is primarily composed of adipocytes (∗; inside the mass lumen, ★; outside the mass, H&E). (B‐1, B‐2) The innermost lumen of the mass is lined by endothelial cells, underlain by a thin layer of elastin and collagen fibers (B‐1; H&E, B‐2; E&M). (B‐3) The endothelial cells lining the innermost lumen of the mass show strong immunoreactivity for CD31. (C‐1) The mass is composed of adipocytes, and no cardiomyocytes are present (H&E). (D‐1, D‐2) A focal area on the external surface of the mass is covered by a layer of collagen (D‐1; H&E, D‐2; E&M).

Histological examination showed that the wall of the mass with an aneurysmal appearance comprised fat cells, whereas cardiomyocytes were absent (Figure 4A‐1,C‐1). The intramural lumen of the mass was covered by endothelial cells, beneath which was a thin layer of elastin and collagen fibers (Figure 4B‐1–B‐3). The epicardium was preserved because the outer surface of the mass was covered with collagen, elastic fibers, and sparse blood vessels (Figure 4D‐1,D‐2).

Based on these pathological findings, we concluded that the mass was a lipoma in the form of an RV aneurysm. The patient recovered without any complications and was discharged on postoperative day 14. A postoperative RV catheterization demonstrated a significant reduction in RVEDP from 25 to 4 mmHg.

## 3. Discussion

Lipomas can originate from any layer of the heart but have a predilection for the visceral and parietal pericardium. They can occur throughout the heart; however, the most common chambers involved are the RA and left ventricle [3]. Notably, <5% of lipomas originate from the RV. Cardiac lipomas are usually asymptomatic, with most cases being identified incidentally [4]. Generally, tumors with intracavitary extension can lead to outflow obstruction or congestive heart failure, potentially infiltrating the conduction system and causing arrhythmias [5, 6].

Several cases of cardiac lipoma with an aneurysm‐like or pseudoaneurysm‐like morphology have been reported. Kondo et al. [7] reported a large cardiac lipoma with a pseudoaneurysmal appearance. Artemiou et al. [8] described an RV lipoma with a pseudoaneurysmatic appearance that communicated with the ventricle and contributed to progressive enlargement of the central cavitation. Similarly, Song et al. [9] reported an extensive cardiac lipoma associated with an aneurysmal RV, in which imaging showed a cavity communicating with the RV, and surgical treatment included resection of the RV anterior wall aneurysm. These reports show that cardiac lipoma can rarely mimic a ventricular aneurysm or pseudoaneurysm.

Differential diagnoses included an RV pseudoaneurysm and liposarcoma. An RV pseudoaneurysm is considered when the patient has a history of trauma or myocardial infarction [10]. On imaging, liposarcoma may show nonuniformly thick septa and mass‐like nonadipose areas [11]. Although multimodality imaging suggests that the lesion is predominantly composed of adipose tissue and communicated with the RV, multimodality imaging alone is insufficient to determine the microscopic structure of the mass or to exclude other differential diagnoses [12]. Therefore, histopathological examination is essential to establish the final diagnosis and guide management and prognosis [12].

Owing to their rarity, no standardized clinical treatment guidelines for cardiac lipomas exist. In general, large lipomas, especially those associated with symptoms or hemodynamic compromise, are considered candidates for surgical resection [13]. In contrast, smaller tumors without symptoms, hemodynamic effects, or diagnostic uncertainty may be monitored to avoid surgical treatment [14]. A systematic review of cardiac lipomas reported that conservative management may be implemented in patients without symptoms related to cardiac lipoma, although prophylactic resection has also been performed in selected asymptomatic patients [13]. Therefore, management should be individualized according to symptoms, tumor size, anatomical location, morphology, hemodynamic impact, and diagnostic uncertainty. In the present case, the patient did not exhibit symptoms such as dyspnea or edema. However, right‐heart catheterization showed elevated RAP and RVEDP, suggesting early hemodynamic compromise. Moreover, the wall of the RV aneurysm‐like lesion was only a few millimeters thick, raising concern for structural fragility. The lesion also showed an atypical pseudoaneurysm‐like morphology, and the preoperative diagnosis remained uncertain despite the imaging evaluation. Based on these findings, surgical resection was selected after careful consideration.

The mechanism by which an RV lipoma develops an aneurysm‐like appearance remains uncertain. In the present case, immunohistological analysis showed CD31 positivity within the intraluminal lumen of the lipoma and abundant elastic fibers. These findings may suggest the presence of blood vessel‐like structures or changes related to blood flow exposure. Based on these findings, one possible explanation is that a lipoma arising from part of the RV wall gradually expanded, and pressure from the ventricular cavity allowed blood to flow into the lesion, resulting in outward protrusion and an aneurysm‐like configuration. Another possible hypothesis is that neovascular structures formed within the RV lipoma and eventually communicated with the RV cavity. However, these mechanisms remain hypothetical, and the patient had no previous CT or echocardiography records, making it difficult to determine the precise developmental process of the lipoma with an aneurysmal appearance.

This case highlights that RV lipoma, although rare, should be considered in the differential diagnosis of a pseudoaneurysm‐like RV lesion. Even in an asymptomatic patient, surgical resection may be reasonable when the lesion shows atypical morphology and causes hemodynamic changes.

## Author Contributions

Yuta Inoue, Takayoshi Kato, Ryo Fujii, Mizuki Kadota, Natsuko Suzui, Masayuki Sato, Hiroki Ogura, Etsuji Umeda, Osamu Sakai, and Kiyoshi Doi performed the data collection, analysis, and interpretation. Yuta Inoue drafted the manuscript. Takayoshi Kato and Kiyoshi Doi critically revised the manuscript for important intellectual content. All authors approved the submitted version of the manuscript and agreed to be accountable for all aspects of the work.

## Funding

No funding was received for this research.

## Consent

Informed consent for the publication of this case report was obtained from the patient.

## Conflicts of Interest

The authors declare no conflicts of interest.

## Supporting Information

Additional supporting information can be found online in the Supporting Information section.

## Supporting information


**Supporting Information** Video S1: Color doppler transthoracic echocardiography. Color doppler transthoracic echocardiography demonstrated to‐and‐fro flow between the right ventricle and the mass through multiple slit‐like communications, supporting communication between the right ventricular cavity and the lipomatous lesion.

## Data Availability

The data that support the findings of this study are available from the corresponding author upon reasonable request.
